# Etiología de baja visión y ceguera en siete centros de referencia en Colombia entre los años 2012 a 2017*

**DOI:** 10.15649/cuidarte.2036

**Published:** 2022-10-16

**Authors:** Juan Camilo Suárez Escudero, María del Pilar Oviedo Cáceres, Yuliana Llano Naranjo, Johana Arias Uribe, José Daniel Villegas Mesa, María Camila Zapata Vásquez, Jorge Luis Ferreira Morales, Jessica Tatiana Reyes Cisneros, Karen Cano Calle, Sydney Goldfeder de Gracia, Juan Felipe González Franco, Esau Astudillo Valverde

**Affiliations:** 1 . Universidad Pontificia Bolivariana (UPB), Universidad CES e Instituto Tecnológico Metropolitano. Medellin, Colombia. Email: juanca.suarez@upb.edu.co Universidad Pontificia Bolivariana Universidad Pontificia Bolivariana Medellin Colombia juanca.suarez@upb.edu.co; 2 . Universidad Santo Tomás, Bucaramanga, Colombia. Email: maria.oviedo@ustabuca.edu.co Universidad Santo Tomás Universidad Santo Tomás Bucaramanga Colombia maria.oviedo@ustabuca.edu.co; 3 . Universidad Pontificia Bolivariana (UPB). Medellín, Colombia. Email: yuligeme@gmail.com Universidad Pontificia Bolivariana Universidad Pontificia Bolivariana Medellín Colombia yuligeme@gmail.com; 4 . Universidad Pontificia Bolivariana (UPB). Medellín, Colombia. Email: johana.arias@upb.edu.co Universidad Pontificia Bolivariana Universidad Pontificia Bolivariana Medellín Colombia johana.arias@upb.edu.co; 5 . Universidad Pontificia Bolivariana (UPB). Medellín, Colombia. Email: jose.villegasm@upb.edu.co Universidad Pontificia Bolivariana Universidad Pontificia Bolivariana Medellín Colombia jose.villegasm@upb.edu.co; 6 . Universidad Pontificia Bolivariana (UPB). Medellin, Colombia. Email: maria.zapatav@upb.edu.co Universidad Pontificia Bolivariana Universidad Pontificia Bolivariana Medellin Colombia maria.zapatav@upb.edu.co; 7 . Universidad Pontificia Bolivariana (UPB). Medellin, Colombia. Email: jorge.ferreira@upb.edu.co Universidad Pontificia Bolivariana Universidad Pontificia Bolivariana Medellin Colombia jorge.ferreira@upb.edu.co; 8 . Universidad Pontificia Bolivariana (UPB). Medellin, Colombia. Email: jessica.reyes@upb.edu.co Universidad Pontificia Bolivariana Universidad Pontificia Bolivariana Medellin Colombia jessica.reyes@upb.edu.co; 9 . Universidad Pontificia Bolivariana (UPB). Medellin, Colombia. Email: karen.cano@upb.edu.co Universidad Pontificia Bolivariana Universidad Pontificia Bolivariana Medellin Colombia karen.cano@upb.edu.co; 10 . Universidad Pontificia Bolivariana (UPB). Medellin, Colombia. Email: sydney.goldfeder@upb.edu.co Universidad Pontificia Bolivariana Universidad Pontificia Bolivariana Medellin Colombia sydney.goldfeder@upb.edu.co; 11 . Universidad Pontificia Bolivariana (UPB). Medellin, Colombia. Email: juanca.suarez@upb.edu.co Universidad Pontificia Bolivariana Universidad Pontificia Bolivariana Medellin Colombia juanca.suarez@upb.edu.co; 12 . Universidad Pontificia Bolivariana (UPB). Medellin, Colombia. Email: esau.astudillo@upb.edu.co Universidad Pontificia Bolivariana Universidad Pontificia Bolivariana Medellin Colombia esau.astudillo@upb.edu.co

**Keywords:** Ceguera, Baja Visión, Etiologia, Oftalmologia, Estadisticas de Secuelas y Discapacidad, Blindness, Low Vision, Etiology, Ophthalmology, Statistics on Sequelae and Disability, Cegueira, Baixa Visáo, etiologia, Oftalmologia, Estatísticas de Sequelas e Incapacidade

## Abstract

**Introducción:**

La baja visión y la ceguera tienen alta prevalencia mundial, siendo categorias de discapacidad frecuentes en Colombia. Se requieren estudios que caractericen la etiologia de las deficiencias visuales permanentes.

**Objetivo:**

Identificar y caracterizar las diferentes causas de baja visión y ceguera en siete centros de referencia para la población con discapacidad visual en Colombia, atendida entre los años 2012 a 2017 en seis ciudades capitales.

**Materiales y métodos:**

estudio retrospectivo, serie de casos, descriptivo y multicéntrico.

**Resultados:**

Se contó con una muestra de 879 registros de pacientes con discapacidad visual. El 70% (612/879) con baja visión y 30% (267/879) con ceguera. Para todos los grupos de edad es más prevalente la baja visión. La etiologia más frecuente en pacientes con baja visión fue la degeneración macular asociada a la edad (DMAE) (24%, 144/612); en pacientes con ceguera fue el glaucoma (17%, 45/267).

**Discusión:**

Posiblemente en Colombia las causas de baja visión y ceguera van más allá de las cataratas, errores de refracción no corregidos y ceguera infecciosa.

**Conclusiones:**

las etiologias más frecuentes encontradas son condiciones oculares crónicas y diversas, que requieren intervenciones especificas para disminuir su prevalencia y prevenir casos de baja visión y ceguera.

## Abstract (en)

**Introduction:**

Low vision and blindness have high global prevalence, with categories of disability common in Colombia. Studies that characterize the etiology of permanent visual impairments are required.

**Objective:**

To identify and characterize the different causes of low vision and blindness in seven reference centers for the visually impaired population in Colombia, attended between 2012 and 2017 in six capital cities.

**Materials and methods:**

retrospective, case series, descriptive and multicenter study.

**Results:**

A sample of 879 records of visually impaired patients was available. Low vision is more prevalent for all age groups. 70% (612/879) low vision and 30% (267/879) blindness. The most common etiology in patients with low vision was age-related macular degeneration (DMAE) (24%, 144/612); in patients with blindness it was glaucoma (17%, 45/267).

**Discussion:**

Possibly in Colombia the causes of low vision and blindness go beyond cataracts, un corrected refractive errors and infectious blindness.

**Conclusions:**

The most common etiologies found are chronic and diverse eye conditions, which require specific interventions to decrease their prevalence and prevent cases of low vision and blindness.

## Resumo

**Introdujo:**

Baixa visáo e cegueira tém alta prevaléncia global, com categorías de incapacidade comuns na Colombia. Sao necessários estudos que caracterizem a etiologia das deficiencias visuais permanentes. **Objetivo:**Identificar e caracterizar as diferentes causas de baixa visao e cegueira em sete centros de referéncia para a populagáo deficiente visual na Colombia, atendidos entre 2012 e 2017 em seis capitais.

**Materiais e métodos:**

estudo retrospectivo, série de casos, descritivo e multicéntrico.

**Resultados:**

Uma amostra de 879 registros de pacientes com deficiéncia visual estava disponível. 54% (478/879) homens. A baixa visao é mais prevalente para todas as faixas etárias. 70% (612/879) baixa visao e 30% (267/879) cegueira. A etiologia mais comum em pacientes com baixa visáo foi a degenerado macular relacionada a idade (DMAE) (24%, 144/612); em pacientes com cegueira foi glaucoma (17%, 45/267).

**Discussao:**

Possivelmente na Colombia as causas da baixa visáo e cegueira váo além da catarata, erros refrativos náo corrigidos e cegueira infecciosa.

**Condusdes:**

As etiologias mais comuns encontradas sáo condigóes oculares crónicas e diversas, que requerem intervengóes específicas para diminuir sua prevaléncia e prevenir casos de baixa visáo e cegueira.

## Introducción

En el informe mundial sobre discapacidad de 2011, la Organización Mundial de la Salud (OMS) estimó que más de mil millones de personas tenían alguna forma de discapacidad (prevalencia mundial del 15%)^1^^,^^2^. En 2018 la OMS reportó que cerca de 1300 millones de personas viven con alguna forma de deficiencia visual (deficiencia visual leve, moderada y/o grave para ver de cerca o lejos, y ceguera), donde al menos 36 millones de personas tienen ceguera y 217 millones baja visión^3^. La ceguera y la baja visión (discapacidad visual) pueden afectar hasta 2.200 millones de personas, donde al menos mil millones tienen una discapacidad visual prevenible o una situación que no ha sido abordada o detectada^4^. Este panorama es aún más complejo pues para el 2050 el número de personas con discapacidad visual podría triplicarse hasta al menos 115 millones de personas invidentes^3^. Para el año 2020 se estimó, que existían 43 millones de personas con ceguera, y 295 millones de personas experimentaron un deterioro de la visión de moderado a severo, que es el 3.74% de la población mundial, con 37.4 casos por 1000 personas. Para el 2050, debido al envejecimiento, el crecimiento y la urbanización de la población, entre otras, se prevé un aumento considerable dado que se proyectan cifras de 474 millones de personas con discapacidad visual^5^.

La mayoría de las personas con baja visión residen en el sur y este de Asia, con una prevalencia que varía entre el 1,6% y el 3,7%, donde Egipto, Myanmar y Afganistán tienen una prevalencia >28% en la población adulta mayor^3^. Para 2020, en la región de América Central donde se ubica Colombia, se reportaron 34 millones de personas con pérdida de visión, de ellas, 1,3 millones eran ciegas^6^.

Mundialmente las principales causas de deficiencias visuales que conducen a discapacidad visual, son los errores de refracción no corregidos, cataratas, degeneración macular asociada a la edad (DMAE), glaucoma, retinopatía diabética, opacidades corneales y tracoma^3^^,^^7^^,^^8^. En América Latina y el Caribe las cataratas son la causa más común de ceguera, y los errores de refracción no corregidos los más comunes de baja visión^9^. En el sur de América Latina la DMAE, es una causa frecuente de deficiencia visual permanente y discapacidad visual^10^.

Para el caso específico de Colombia, el censo poblacional del 2005 estableció una prevalencia de discapacidad del 6,4%, donde un 43,2% (más de un millón) de colombianos reportaron limitaciones permanentes para ver a pesar de usar lentes o gafas^11^. Por su parte, el censo poblacional del año 2018 reportó una prevalencia nacional de discapacidad del 7,1% (3'065.361 personas), donde las dificultades tipo no poder ver de cerca, de lejos o a su alrededor constituyen el 18,7%^12^. Pese a lo anterior, es necesario mencionar que ninguno de los censos discrimina con detalle la población con baja visión ni con ceguera, y a su vez, tampoco se identifican estudios poblacionales específicos que estimen la prevalencia de discapacidad visual en el país.

La pérdida de la visión, las enfermedades oculares relacionadas con la edad y la discapacidad visual se consideran retos de salud pública, porque además de su alta prevalencia e implicaciones en salud, el deterioro de la visión afecta las oportunidades económicas y educativas, disminuye la calidad de vida y aumenta la probabilidad de muerte^13^^,^^14^. Diferentes estudios reportan las limitaciones que experimentan las personas con discapacidad visual para realizar actividades diarias y tareas que requieren de la visión, como el estudio y el trabajo; lo cual restringe la participación de las personas, la capacidad de ejercer sus derechos, la generación de ingresos, la toma de decisiones y el acceso a espacios tecnológicos y arquitectónicos, entre otras actividades^15^^,^^16^.

En este sentido y dadas las implicaciones de la discapacidad visual en la vida cotidiana de las personas, la OMS ha promovido la iniciativa VISIÓN 2020 y el Universal Eye Health, plan de acción global 2014 2019^17^, como estrategias para el desarrollo de acciones que permitan implementar programas de prevención de la ceguera a nivel nacional y regional entre las que se incluye la necesidad de generar evidencia que muestre la prevalencia y etiología de la discapacidad visual^18^. Pese a lo anterior, Colombia carece de estudios multicéntricos y/o poblacionales sobre la etiología de discapacidad visual, que permita acercarse a una comprensión del fenómeno en términos asistenciales y de salud pública, y de esta manera dar herramientas para la planificación en salud.

Al momento de la ejecución de esta investigación, solo se rastrean dos estudios en el contexto colombiano, uno realizado en el 2015 en la ciudad de Medellín donde se documentaron etiologías como miopía patológica, neuropatía óptica no glaucomatosa, glaucoma y DMAE^19^ y otro en el Departamento de Santander que reportó como principal causa de ceguera las cataratas, alteraciones del segmento posterior, opacidades corneales y glaucoma^20^. Si bien estos estudios son muy pertinentes, se requieren más estudios epidemiológicos y clínicos sobre la discapacidad visual debido a que la información disponible es limitada^9^: se carece de estudios poblacionales que caractericen clínica y socio demográficamente la población con deficiencias visuales permanentes en Colombia y Latinoamérica; las posibles causas de las deficiencias visuales provienen de estudios de alcance local en dos o tres ciudades de Colombia; varios estudios se centran en las causas reversibles como las ametropías y catarata, y no en explorar otras etiologías de índole neuro oftalmológico y enfermedades oculares crónicas; y en el caso colombiano se desconocen las causas más frecuentes de ceguera y baja visión en la población.

Por tanto, el objetivo de este estudio fue identificar y caracterizar las diferentes causas de baja visión y ceguera permanente (no reversible) en siete centros de referencia para la población con discapacidad visual en Colombia, para adquirir datos útiles que mejoren la comprensión de las condiciones de salud relacionadas con el espectro funcional de la discapacidad visual, y aproximarse al verdadero impacto de las enfermedades oculares crónicas en el origen de las deficiencias visuales permanentes.

## Materiales y métodos

Estudio retrospectivo, tipo serie de casos, descriptivo y multicéntrico realizado en siete instituciones y centros de salud visual colombianas. Se diseñó y utilizó un formato de recolección de información con variables sociodemográficas (edad, sexo y lugar de origen) y variables clínicas (comorbilidades no oftalmológicas, etiología causante de la baja visión o ceguera, agudeza visual mejor corregida, categoría de discapacidad visual, informe de suministro de ayudas ópticas y no ópticas, y proceso de rehabilitación) registrados en historia clínica. Como fuente de datos se tomaron los registros e historias clínicas de pacientes con discapacidad visual atendidos entre los años 2012 a 2017, en siete centros y/o instituciones de salud con servicios de oftalmología y/o discapacidad visual ubicados en seis ciudades de Colombia: Bogotá, Bucaramanga, Cali, Cartagena, Medellín y Pereira. Estas instituciones se identificaron a partir de la Red Nacional de Discapacidad Visual; grupo colaborativo colombiano conformado por escuelas de optometría y medicina, así como centros e instituciones que ofrecen servicios de salud para personas con discapacidad visual y rehabilitación de la visión.

Se utilizaron las categorías de la OMS para clasificar la gravedad de las deficiencias visuales dadas en la clasificación internacional de enfermedades CIE-10R y CIE-11, a partir de rangos de agudeza visual (AV): baja visión abarca una AV mejor corregida peor que 20/60 pero igual o mejor que 20/400 en el mejor ojo o pérdida de campo visual correspondiente a menos de 20° en el mejor ojo con la mejor corrección posible; y ceguera consiste en una AV <20/400 (<3/60). Se aclara que la baja visión agrupa dos categorías a saber: deficiencias visuales moderadas (AV de <6/18 o <20/60) y graves (AV de <3/60 o <20/200), en el mejor ojo con la mejor corrección posible ^3^^,^^7^^,^^21^.

Entre mayo de 2017 y enero de 2018, se realizaron una a dos visitas programadas en los siete centros/ instituciones de salud participantes con servicio de oftalmología y/o discapacidad visual. Cada centro/ institución fue visitado por dos a tres miembros del equipo de investigación (un profesional de la salud visual tipo oftalmólogo u optómetra especializado en baja visión, en compañía de uno o dos asistentes de investigación) para revisar las fuentes de datos y aplicar el formato de recolección de información del estudio.

Se llevó a cabo un proceso de estandarización interna del equipo de investigación (conformado por tres médicos, dos de ellos residentes de oftalmología, dos optómetras con entrenamiento en baja visión, y uno con maestría en salud pública, y siete estudiantes de medicina) en términos de lectura y extracción homogénea de los datos de las historias clínicas, unificación del diligenciamiento del formato de recolección y aplicación de los criterios de la OMS de baja visión y ceguera según AV, previo a las visitas programadas a cada una de las instituciones/centros del estudio.

Los criterios de inclusión para los casos fueron los siguientes: a) historias clínicas de pacientes que cumplan criterios de discapacidad visual según la definición de la OMS (ceguera y baja visión, en el mejor ojo, después de la mejor corrección posible); b) donde al menos el sexo, la AV y el diagnóstico principal fueran identificados en los registros de historia clínica. Por su parte los criterios de exclusión fueron: diagnóstico principal visual no aclarado o registros incompletos en cuanto a examen visual. Los casos fueron obtenidos de centros o instituciones de salud con servicios de oftalmología y/o discapacidad visual.

Se utilizó un muestreo no probabilístico secundario a las dificultades en el acceso a la población dentro del mismo sistema de salud donde hay subregistro y pobre codificación diagnóstica mediante CIE-10R de las deficiencias visuales. La muestra se obtuvo mediante criterio de autoridad puesto que los profesionales de salud llamados a identificar y atender este tipo de población son oftalmólogos y optómetras^22^^,^^23^.

Investigación sin riesgo, basada en lectura, revisión y extracción de información a partir de historias/ registros clínicos. El estudio fue aprobado por el Comité de Investigación y Ética de la Escuela de Ciencias de la Salud, Universidad Pontificia Bolivariana (Medellín, Colombia) y el Comité de Ética de cada una de las instituciones participantes.

En las variables cualitativas se obtuvo distribución de frecuencias para cada categoría. En variables cuantitativas se evaluó normalidad; y en aquellas sin distribución normal se obtuvo mediana, cuartil 1 y cuartil 3. Los resultados se mostrarán de dos formas: resultados globales agrupados por baja visión y ceguera, y las dos etiologías con mayor frecuencia por grupo etario (menores de 15 años, edad entre 15 a 49 años, y edad >50 años) para indicar la distribución y características de la discapacidad visual acorde al ciclo de vida. La base de datos fue diseñada en Excel Office 365. Se utilizó el paquete estadístico SPSS 21 y Epidat 3.1. La base de datos tiene acceso público en Mendeley Data^24^.

Los pacientes del estudio fueron clasificados en dos desenlaces excluyentes a partir de la deficiencia visual por AV a saber: baja visión o ceguera. Para el cálculo de los odds ratio (OR) se construyeron tablas de contingencia para estudio transversal en el programa Epidat 3.1 donde se evaluó la asociación entre las variables demográficas y clínicas, y la presencia de ceguera. Se seleccionó este desenlace por ser el más grave dentro del espectro de la discapacidad visual (ausencia total de visión). Por lo tanto, los factores de riesgo encontrados hacen relación a la probabilidad de tener ceguera; en algunos OR cuyo resultado indica ser un factor protector, se debe a que la presencia de esa exposición aumenta la probabilidad de baja visión en lugar de ceguera. Como variables de exposición se tomaron los diferentes grupos de edad, lugar de origen, principales comorbilidades y la presencia de las etiologías más frecuentes encontradas en el estudio. Para el caso de la edad la variable está clasificada en tres grupos (menor a 15 años, entre 15 y 49 años, y >50 años), de los cuales se tomó como referencia para el cálculo de los OR el grupo de edad entre 15 y 49 años debido a que es el grupo donde se observaría menor frecuencia e impacto de las causas de deficiencias visuales permanentes de origen congénito y relacionadas con el envejecimiento. Cada OR se acompaña por el correspondiente intervalo de confianza del 95%, y el valor p obtenido a partir de la prueba Chi cuadrado de asociación sin corrección.

## Resultados

Se revisaron 1628 historias clínicas entre las siete instituciones/centros incluidas, obteniendo una muestra de 879 registros por criterios de elegibilidad, con la siguiente distribución de instituciones y pacientes por ciudad: Cali (suroeste de Colombia: una institución) 28% de los pacientes (250/879); Medellín (centro, noroeste de Colombia: dos instituciones) 24% (207/879); Bucaramanga (noreste de Colombia: una institución) 21% (183/879); Bogotá (centro de Colombia: una institución) 18% (156/879); Cartagena (noroeste de Colombia: una institución) 7% (62/879); y Pereira (centro, occidente de Colombia: una institución) 2% (21/879). El 54% (478/879) fueron hombres. La muestra tuvo una distribución bimodal de la edad: menores de edad (<18 años) 19% (168/864), y edad >18 años 81% (696/864). En 15 historias clínicas (15/879) no fue registrada la edad y en otras cinco (5/879) no se registró el lugar de origen de la persona. En los menores de 18 años una mediana de edad de 9 años (Q1: 6; Q3: 12), y en los mayores de 18 años una mediana de 54 años (Q1: 35; Q3: 73,5). La Tabla 1 muestra las características demográficas y clínicas de los pacientes según deficiencia visual.

**Tabla 1:** Características demográficas y clínicas de los pacientes por categoría de discapacidad visual. n (%)

Características	Total	Ceguera	Baja visión	valor p*	OR	IC (95%)
Grupos de edad, n=864**	864	261	603			
Menor a 15 años	145 (16,8)	54 (20,7)	91 (15,1)	0,683	1,09	0,71-1,67
Entre 15 y 49 años	250 (28,9)	88 (33,7)	162 (26,9)			1
Mayor o igual a 50 años	469 (54,3)	119(45,6)	350 (58,0)	0,005	0,62	0,44-0,87
Lugar de origen, n=874**	874	265	609			
Área urbana	790 (90,4)	238 (89,8)	552 (90,6)	0,702	0,91	0,56-1,47
Área rural	84 (9,6)	27 (10,2)	57 (9,4)			
Principales comorbilidades, n=551	551	170	381			
Hipertensión arterial	228 (41,4)	50 (29,4)	178 (46,7)	0,001	0,47	0,32 - 0,69
Enfermedad neurológica	90 (16,3)	46 (27,1)	44 (11,5)	0,000	2,84	1,79 - 4,50
Enfermedad diabética	89 (16,1)	19 (11,2)	70 (18,4)	0,034	0,55	0,32 - 0,96
Dislipidemia	46 (8,3)	12 (7,1)	34 (8,92)	0,464	0,77	0,39 - 1,53
Enfermedad cardiovascular	28 (5,1)	6 (3,5)	22 (5,8)	0,267	0,59	0,23 - 1,50
Enfermedad pulmonar	22 (3,4)	4 (2,4)	18 (4,7)	0,189	0,48	0,16 - 1,45
Enfermedad hematológica	6 (1,1)	3 (1,8)	3 (0,8)	0,307	2,26	0,45 - 11,33
Traumatismo cráneo encefálico	10 (1,8)	7 (4,1)	3 (0,8)	0,006	5,41	1,38 - 21,18
Enfermedad neonatal	32 (5,8)	23 (13,5)	9 (2,4)	0,000	6,46	2,92 - 14,3
Etiologías más frecuentes del déficit visual, n=879	879	267	612			
Degeneración macular asociada a la edad	160 (18,2)	16 (5,9)	144 (23,5)	0,000	0,20	0,12 - 0,35
Glaucoma (incluye congénito)	137 (15,6)	45 (16,8)	92 (15,0)	0,493	1,14	0,77 - 1,69
Neuropatía óptica no glaucomatosa	60 (6,8)	22 (8,2)	38 (6,2)	0,272	1,35	0,78 - 2,34
Retinitis pigmentosa	54 (6,1)	16 (5,9)	38 (6,2)	0,902	0,96	0,52 - 1,75
Toxoplasmosis ocular	47 (5,3)	12 (4,5)	35 (5,7)	0,458	0,77	0,39 - 1,51
Miopía patológica	46 (5,2)	10 (3,7)	36 (5,8)	0,190	0,62	0,30 - 1,27
Malformaciones congénitas***	43 (4,9)	22 (8,2)	21 (3,4)	0,002	2,52	1,36 - 4,67
Retinopatía del prematuro	38 (4,3)	25 (9,3)	13 (2,1)	0,000	4,76	2,39 - 9,45
Retinopatía diabética	38 (4,3)	10 (3,7)	28 (4,5)	0,578	0,81	0,38 - 1,69
Distrofias****	38 (4,3)	6(2,2)	32 (5,2)	0,045	0,41	0,17 - 1,00

Los porcentajes se muestran por columnas y no por filas.

*Chi cuadrado de asociación**.**

** en 15 historias clínicas no fue registrada la edad en cinco historias clínicas no se registró el lugar de origen de la persona, por lo que el n para algunos cálculos varia.

***del nervio óptico, coriorretiniana de se mento anterior. **** de conos bastones, hereditaria de retina, macular hereditaria de córnea.

Para todos los grupos de edad es más prevalente la baja visión que la ceguera y a su vez las dos condiciones son más frecuentes a medida que aumenta la edad. Se encontró que las personas con 50 o más años tienen menor probabilidad de tener ceguera, en comparación con el grupo de 15 a 49 años. Sin embargo, cuando se comparan estos dos grupos de edad, frente al riesgo de tener baja visión, se observa que el grupo de 50 o más años tiene mayor probabilidad de tener baja visión (valor p: 0,005. OR: 1,59. IC 95%: 1,14-2,22). No se encontró asociación estadísticamente significativa por el lugar de origen urbana o rural de los pacientes. Se identificaron tres comorbilidades que se comportan como factores de riesgo para la presencia de ceguera: enfermedad neurológica, traumatismo cráneo encefálico (TEC) y enfermedad neonatal. De las cuales el TEC y la enfermedad neonatal son las que mayor fuerza de asociación mostraron con el desenlace. No obstante comorbilidades como la hipertensión arterial y enfermedad diabética se comportaron como factores protectores frente al riesgo de ceguera, pero ambas comorbilidades frente al desenlace de baja visión se comportan como factores de riesgo: hipertensión arterial (valor p: 0,000. OR: 2,10. IC 95%: 1,42-3,09) y enfermedad diabética (valor p: 0,034. OR: 1,78. IC 95%: 1,03-3,07). Las etiologías retinopatía del prematuro y malformaciones congénitas poseen asociación estadística como factores de riesgo para ceguera, pero la degeneración macular asociada a la edad se encontró como un factor protector. Pero esta última frente al desenlace de baja visión se comporta como un factor de riesgo (valor p: 0,034. OR: 4,82. IC 95%: 2,81-8,27). Ninguna de las demás comorbilidades y etiologías presentó diferencias estadísticamente significativas.

En cuanto a la categoría de la discapacidad visual se encontró que el 70% (612/879) corresponde a pacientes con baja visión y el 30% (267/879) a ceguera. La Figura 1 muestra la distribución de las categorías de deficiencia visual de los pacientes.

**Grafica 1:**
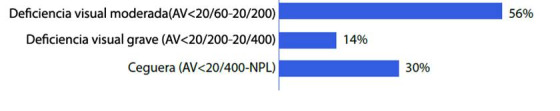
Distribución de las categorías de deficiencia visual. AV: agudeza visual. NPL: no percepción de luz.

Se logró reconocer y categorizar 41 etiologías responsables fisiopatológicamente de producir la deficiencia visual permanente en rango baja visión o ceguera. Las etiologías con mayor frecuencia en toda la muestra fueron DMAE, glaucoma (incluye congénito), neuropatía óptica no glaucomatosa y retinitis pigmentosa. En la Tabla 2 se muestra la distribución de las diez etiologías con mayor frecuencia identificadas, y agrupadas por baja visión y ceguera, según el grupo de edad.

**Tabla 2:** Distribución de las diez etiologías más frecuentes por categoría de discapacidad visual y por grupo de edad

		Baja visión*		Ceguera**		
Etiologías	Menor a 15 años (n=91)	Entre 15 y 49 años (n=162)	Mayor o igual a 50 años (n=350)	Menor a 15 años (n=54)	Entre 15 y 49 años (n= 88)	Mayor o igual a 50 años (n=119)
	n (%)	n (%)	n (%)	n (%)	n (%)	n (%)
Degeneración macular asociada a la edad	0 (0)	25 (15,4)	119 (34)	0 (0)	5(5,7)	11 (9,2)
Glaucoma (incluye congénito)	14 (15,4)	18 (11,1)	60 (17,1)	7 (13)	12 (13,6)	26 (21,8)
Neuropatía óptica no glaucomatosa	8 (8,8)	10 (11,7)	20 (5,7)	5 (9,3)	7 (8)	10 (8,4)
Retinitis pigmentosa	3 (3,3)	15 (9,3)	20 (5,7)	2 (3,7)	7 (8)	7 (5,9)
Toxoplasmosis ocular	15 (16,5)	13 (8)	7(2)	5 (9,3)	3 (3,4)	4 (3,4)
Miopía patológica	6 (6,6)	13 (8)	17 (4,9)	1 (1,9)	3 (3,4)	6 (5)
Malformaciones congénitas***	7 (7,7)	6 (3,7)	8 (2,3)	7 (13)	8 (9,1)	7 (5,9)
Retinopatía del prematuro	8 (8,8)	4 (2,4)	2 (0,6)	14 (24,1)	7 (8)	4 (3,4)
Retinopatía diabética	3 (3,3)	3 (1,9)	22 (6,3)	0 (0)	2 (2,3)	8 (6,7)
Distrofias****	5 (5,5)	14 (8,6)	13 (3,7)	0 (0)	3 (3,4)	3 (2,5)

Los porcentajes se muestran por tanto, columnas y no por fila pacientes con baja visión no se registró la edad en historia clínica, por lo se muestran datos de 603 de 612 pacientes con baja visión. **en seis pacientes con ceguera no se registró la edad en historia clínica, por lo tanto, se muestran los datos de 261 de 267 pacientes con ceguera. ***del nervio óptico, coriorretiniana y de segmento anterior. **** de conos bastones, hereditaria de retina, macular hereditaria de córnea.

En los pacientes con baja visión (612/879) las dos etiologías más frecuentes fueron la DMAE con el 24% (144/612) y el glaucoma (incluye congénito) con el 15% (92/612) de frecuencia. En las personas con ceguera (267/879) las dos etiologías más frecuentes fueron el glaucoma (incluye congénito) con un 17% (45/267) y la ROP 9% (25/267).

Otras etiologías clásicas (por literatura) como catarata (incluye catarata senil y catarata congénita), albinismo, trauma ocular y tumores (incluyendo retinoblastoma) tuvieron frecuencias menores al 3% en toda la muestra. Por su parte etiologías infecciosas causantes de deficiencias visuales, diferentes a toxoplasmosis, fueron un caso de retinitis por citomegalovirus, un caso de retinopatía por VIH y un caso de Toxocara canis. La frecuencia de trastornos en la vía visual posterior (vía visual intracerebral y corteza cerebral) fue del 2% (20/879) en toda la muestra.

Las etiologías más frecuentes de baja visión y ceguera en menores de 15 años fueron la ROP con una frecuencia del 15% (22/145), el glaucoma (incluye congénito) con un 14,5% (21/145) y la toxoplasmosis ocular con un 14% (20/145). En el grupo etario de 15 a 49 años y >50 años las dos etiologías más frecuentes fueron la DMAE y el glaucoma.

Datos sobre procesos de rehabilitación de la visión se documentaron en las historias clínicas del 58% (509/879) de los pacientes. El registro sobre suministro de ayudas ópticas y no ópticas se identificó en el 56% (496/879) y el 35% (305/879) de los pacientes respectivamente.

## Discusión

Este es el primer estudio multicéntrico con aproximación nacional, sobre la etiología de baja visión y ceguera permanente en Colombia, realizado a través de información recolectada en varias instituciones y centros de referencia que atienden a esta población. Se utilizó una muestra no probabilística, secundario a los desafíos de la población para acceder al sistema de salud, existencia de subregistro y poca sensibilización en la temática por parte de los profesionales de salud, aspectos que son soportados en la investigación de Oviedo y col^25^^,^^26^. Existió variación de la disponibilidad de datos entre instituciones y ciudades del estudio.

En la muestra obtenida, se identificó que el 70% de los pacientes corresponden a baja visión y el 30% a ceguera. Estos resultados son coincidentes con la estimación mundial que indica que que la población con discapacidad visual se distribuye en 20% con ceguera y 80% baja visión^26^ y a su vez con lo reportado en Colombia en 2006, donde se reportó un millón cien mil personas con discapacidad visual, distribuidas en 80% con baja visión y 20% con ceguera^27^.

En el presente estudio, para todos los grupos de edad fue más prevalente la baja visión que la ceguera y a su vez las dos condiciones son más frecuentes a medida que aumenta la edad, (el 53% de la muestra corresponde a personas mayores de 50 años; resultado similar a los datos reportados mundialmente, que señalan que las principales etiologías de deficiencias visuales permanentes están asociadas con la edad, debido a que el 86% de las personas con ceguera y el 80% de las personas con baja visión tienen más de 50 años^4^. Lo anterior se constituye en un elemento importante en términos de salud pública dada la tendencia de envejecimiento de la población, lo que implica que más personas estarán en riesgo de sufrir discapacidad visual por enfermedades oculares crónicas^28^. Colombia experimenta un proceso de envejecimiento poblacional en el que se proyecta que para 2050 la esperanza de vida aumente a 83,4 años para las mujeres y 77,5 años para los hombres, según informes de la Misión Colombia Envejece 1985-2050, elevando así la carga de enfermedades oculares crónicas^29^.

El menor riesgo observado de ceguera en el grupo con edad de 50 o más años, en comparación con los de 15 a 49 años, podría deberse a que este grupo por ser identificado como de mayor riesgo de adquirir discapacidad en general, es priorizado en los servicios de tamizaje, tratamiento y seguimiento oftalmológico más oportuno en cuanto a enfermedades oculares crónicas, con el objetivo de evitar llegar a ceguera, o prolongar más la historia natural de la enfermedad en categoría baja visión. Sin embargo, dicho grupo posee mayor riesgo de baja visión por su edad, pero se podría estar viendo el efecto de las intervenciones para que la baja visión no progrese a ceguera. Una explicación similar a la anterior podría darse a la asociación observada entre hipertensión arterial y enfermedad diabética, y el riesgo de ceguera: donde el menor riesgo observado con estas comorbilidades puede deberse al manejo y seguimiento médico más frecuente dentro de los programas de prevención secundaria que existen en el sistema de salud.

La distribución por sexo fue algo mayor en hombres que en mujeres (54% vs 46%) en toda la muestra, dato que contrasta con los hallazgos reportados en otros estudios tanto nacionales como internacionales, en los cuales se identifica de manera sistemática que los trastornos de la visión afectan a las mujeres de manera desproporcionada en comparación con los hombres^30^^-^^33^. A nivel internacional, dos tercios de la ceguera y la discapacidad visual se producen en las mujeres. Esta relación se mantiene (aunque por diferentes motivos) para prácticamente todas las condiciones de ceguera prevenibles y tratables en el mundo, incluidas la catarata y el tracoma en particular^33^.

En cuanto a la etiología, nuestro estudio identificó la DMAE y al glaucoma como las dos etiologías más frecuentes en toda la muestra de pacientes incluyendo al grupo de pacientes con baja visión y al grupo de pacientes con edad >50 años; y nuevamente el glaucoma junto a la ROP fueron las más frecuentes en el grupo de pacientes con ceguera. Según informes internacionales sobre personas con discapacidad visual, las principales etiologías son los errores refractivos no corregidos y las cataratas como causas reversibles, seguidas del glaucoma y la DMAE como causas irreversibles^3,5^. La literatura indica que las cataratas continúan siendo la principal etiología de la discapacidad visual en América Latina (donde presumiblemente se habían abordado aquellas formas tratables de discapacidad visual como defectos de refracción no corregidos y cataratas); sin embargo, se ha observado una tendencia decreciente al comparar los hallazgos de 1990 a 2010^34^.

Por otro lado el hallazgo de este estudio sobre glaucoma y ROP como las etiologías más frecuentes de ceguera, contrastan con informes internacionales que identifican a los errores refractivos no corregidos, cataratas no operadas y glaucoma como las principales etiologías de ceguera^4,(^^35^. Frente a lo anterior se debe tener presente que nuestro estudio se basó en estimar las principales etiologías de discapacidad visual en pacientes con la mejor AV corregida, y con deficiencia visual permanente, donde varios ya habían recibido cirugía de cataratas y corrección refractiva y hacen parte de instituciones/ centros de manejo y rehabilitación de baja visión y ceguera.

La asociación de la DMAE como factor protector frente a ceguera, pero factor de riesgo con baja visión, se podría explicar por su carácter crónico y progresivo produciendo inicialmente deficiencias visuales en rango baja visión, pero con el tiempo y en ausencia de manejo y seguimiento médico resultaría en ceguera. De igual manera, el manejo y seguimiento por oftalmología y especialistas en retina que requieren estos pacientes busca modificar la historia natural de la DMAE, ralentizando o evitando su progresión a ceguera. Mundialmente la DMAE es la principal causa de ceguera irreversible en población adulta de países industrializados occidentales y en población caucásica^36^^,^^37^. En estadios avanzados de la enfermedad, su prevalencia aumenta del 0,2% en personas de 55 a 64 años al 13,1% en los mayores de 85 años; además, es una causa importante de deterioro visual en adultos mayores^38^. Un estudio australiano reportó que la DMAE es la principal causa de discapacidad visual en el 40% de los casos en personas de 70 años^39^. Wong ha estimado que para 2020, la cantidad de personas con DMAE en todo el mundo será de aproximadamente 200 millones y puede aumentar a 300 millones para 2040^40^. La DMAE como condición ocular crónica es capaz de producir baja visión y ceguera.

En el presente estudio, el glaucoma, incluido el glaucoma congénito, se identificó como la causa más frecuente en los pacientes con ceguera y la segunda causa más frecuente en los pacientes con baja visión (pero no se encontró asociación estadística). Se considera que el glaucoma es la principal causa de ceguera irreversible en el mundo, con una prevalencia estimada de 64 millones de personas afectadas en 2013 (alrededor del 3,5%); se estima que estas cifras aumentarán a aproximadamente 112 millones para 2040^41^. La prevalencia más alta se ha identificado en países africanos y latinoamericanos con 4,2% a 4,5% y 3,4% a 3,65% respectivamente, y la prevalencia más baja se ha identificado en Asia y Europa^42^.

En el grupo etario de 15 a 49 años, la retinosis pigmentaria y neuropatía óptica no glaucomatosa fueron otras etiologías frecuentes, con más casos de baja visión que de ceguera, ocupando el tercer y cuarto lugar en frecuencia en toda la muestra. En un estudio realizado en Kuwait, que analizó las causas de discapacidad visual en 412 pacientes, se informó que la retinosis pigmentaria es la principal etiología de la ceguera, seguida de anomalías congénitas y atrofia óptica; y en personas <20 años, la tasa de atrofia óptica aumentó, seguida de malformaciones congénitas, retinosis pigmentaria y retinopatía del prematuro^43^. La neuropatía óptica no glaucomatosa es una categoría que agrupa las neuropatías ópticas de etiologías distintas al glaucoma, como las neuropatías ópticas isquémicas anterior y posterior, tumores intracraneales, neuritis óptica, entre otras. Un estudio realizado en Nigeria caracterizó a 193 pacientes con baja visión y reportó la atrofia óptica no glaucomatosa como la tercera etiología más común entre pacientes con baja visión^44^.

En el grupo de niños y adolescentes con edad <15 años, la ROP y la toxoplasmosis ocular fueron las dos etiologías más frecuentes, con más casos de baja visión para la toxoplasmosis ocular y más casos de ceguera para la ROP. La toxoplasmosis ocular es una infección que potencialmente puede resultar en pérdida de la visión. Se estima una prevalencia mundial de toxoplasmosis ocular del 2%^45^, existiendo reportes en países en vías de desarrollo que su prevalencia probablemente esté subestimada^46^. Un estudio que comparó niños con toxoplasmosis congénita en Brasil y Europa, entre 1992 y 2003, reportó diferencias en el curso clínico (retinocoroiditis durante el primer año de vida y pérdida severa de la visión por múltiples lesiones localizadas en el polo posterior)^45^^,^^47^. En Colombia se han reportado diferencias asociadas al parásito y huésped, que describen las cepas de Toxoplasma gondii como más virulentas, con un curso clínico más complicado, lesiones más grandes, mayor grado de inflamación, afectación macular, afectación bilateral y recurrencias^48^.

Por su parte, la ROP se considera la principal causa de ceguera infantil en todo el mundo^49^, donde su incidencia y gravedad aumentan al disminuir la edad gestacional y el peso al nacer^50^. La prevalencia es del 5% al 8% en los países desarrollados y aproximadamente del 30% en los países en desarrollo^51^. En Colombia, un estudio realizado en Medellín, entre 2003 y 2008 reportó una prevalencia de ROP del 18,2%^52^; y otro estudio realizado en Colombia en 2006 caracterizó a 124 niños con ceguera, informando que el 33,8% de los casos eran atribuibles a ROP^53^. La prevalencia de ceguera por ROP varía entre diferentes regiones: en los países desarrollados se informa que es una causa de ceguera infantil entre el 3% y 11%, y en América Latina, contribuye al 4% y 38%^51^^,^^54^. En este contexto, es importante mencionar que desde 2008, Colombia cuenta con lineamientos y protocolos nacionales para prevenir, diagnosticar y tratar la ROP, además de los lineamientos técnicos de los programas del plan canguro, que tienen como objetivo disminuir el número de niños con ceguera causada por ROP^55^.

Los resultados del presente estudio sugieren que la ROP y las malformaciones congénitas del sistema visual son factores de riesgo de ceguera y no de baja visión, resultado que podría estar en relación con una mayor deficiencia tanto estructural como funcional en retina en el caso de ROP y de varias estructuras visuales en el caso de malformaciones que llevarían precozmente a AV menores a 20/400 hasta no percepción de luz o pérdida total de la visión.

Adicionalmente se encontró que las personas con deficiencias visuales permanentes, que presenten comorbilidades neurológicas, TEC o enfermedad neonatal tienen mayor riesgo de que el déficit visual este en rango ceguera. Esto se podría explicar por la magnitud fisiopatológica de estas entidades en los componentes centrales del sistema visual y el compromiso de varias funciones de la visión.

Son limitaciones del estudio la disponibilidad y calidad de información en los registros e historias clínicas accedidas (donde solo el 54% de las historias revisadas inicialmente conformó la muestra del estudio), no fue posible contar con un muestreo probabilístico puesto que no hay listas de pacientes con discapacidad visual en el sistema de salud, hay dificultades de acceso por parte de la población con baja visión o ceguera a atenciones en salud en centros especializados, y además existe subregistro y dificultades en la utilización y codificación diagnóstica de las deficiencias visuales mediante CIE- 10R, CIE-11 y Clasificación Internacional del Funcionamiento, Salud y Discapacidad (CIF-2001). No obstante el diseño del estudio permitió acceder a varios centros de salud visual y conformar una muestra amplia de personas con baja visión y ceguera en Colombia que permite una aproximación del comportamiento de las deficiencias visuales permanentes por etapa del ciclo de vida, y más allá de causas clásicas como catarata y ametropías no corregidas; adicionalmente se utilizó un formato de recolección de información estandarizado por el equipo de investigadores para una recolección uniforme de la información necesaria para dar respuesta al propósito del estudio. Se sugiere para futuros estudios contar con los reportes poblacionales del registro de localización y caracterización de población con discapacidad (RLCPD) a partir del proceso oficial de certificación de discapacidad direccionado por la resolución 113 del año 2020 (proceso que utiliza la CIF-2001 e inició en el año 2020 y 2021), y mejorar el proceso de codificación diagnóstica de las deficiencias visuales para poder identificar de mejor manera las personas con deficiencias visuales permanentes y discapacidad visual dentro del sistema de salud.

Finalmente, otras etiologías identificadas en la muestra de este estudio fueron la miopía patológica, malformaciones congénitas, retinopatía diabética y distrofias. De estas cuatro entidades la miopía patológica y la retinopatía diabética poseen mayor documentación en cuanto a baja visión y ceguera. La miopía patológica se considera una causa importante de ceguera legal y baja visión en todo el mundo, particularmente en países asiáticos, reportándose una prevalencia de miopía alta en población hispana del 2,4%^56^^,^^57^. La retinopatía diabética se considera la tercera causa común de baja visión y ceguera legal irreversible en el mundo y la primera causa común en la población económicamente activa^58^.

## Conclusiones

Posiblemente en Colombia las causas de baja visión y ceguera van más allá de las cataratas, errores de refracción no corregidos y ceguera infecciosa (excepto la toxoplasmosis). Las etiologías más frecuentes identificadas en la población de estudio con baja visión fueron la DMAE y el glaucoma, y en ceguera fueron el glaucoma y la ROP, encontrándose otras etiologías con novedad en el contexto de baja visión y ceguera en Colombia (como neuropatía óptica no glaucomatosa y retinosis pigmentaria). Se observaron asociaciones estadísticamente significativas entre población con edad >50 años y baja visión; comorbilidades tipo enfermedad neurológica, TEC y enfermedad neonatal con ceguera; hipertensión arterial y enfermedad diabética con baja visión; ROP y malformaciones congénitas del sistema visual con ceguera; y DMAE con baja visión.

Considerando el envejecimiento de la población en Colombia y el concepto de discapacidad visual relacionada con la edad, se requieren intervenciones específicas y esfuerzos localizados para disminuir su prevalencia y prevenir la posible incidencia de baja visión y ceguera debido a enfermedades oculares crónicas, aspectos que se identificaron en este estudio, como importantes para la presentación de discapacidad visual. Es necesario identificar nuevas estrategias para abordar los desafíos relacionados con la rápida aparición de afecciones oculares crónicas no transmisibles. Por lo tanto, es importante mejorar la disponibilidad de los servicios de atención, garantizando su accesibilidad, asequibilidad y calidad como un mecanismo que puede contribuir a la disminución de la discapacidad visual evitable.

Continúa siendo crítico contar con servicios de rehabilitación para optimizar el funcionamiento en la vida diaria, por tanto, los gobiernos deben establecer políticas nacionales precisas de cobertura de rehabilitación de la salud para las principales causas de baja visión y ceguera basadas en investigaciones epidemiológicas locales. Es necesario continuar con el proceso de fortalecimiento de la atención ocular desde los primeros niveles de atención en el sistema de salud que permita hacer frente a las principales causas de discapacidad visual reportadas en el presente estudio, para brindar una intervención oportuna para prevenir la ceguera y tratar la baja visión.
